# Associations between usual source of care characteristics and health outcomes in diabetes mellitus: a focus on medication adherence and healthy behaviors in Korea

**DOI:** 10.4178/epih.e2025063

**Published:** 2025-12-02

**Authors:** Seung Eun Lee, Chul-Woung Kim, Ji Eun Bae, Jee Hyun Choi

**Affiliations:** 1 Center for Tobacco Control Research, Chungnam National University, Daejeon, Korea; 2 Department of Preventive Medicine and Public Health, Chungnam National University College of Medicine, Research Institute for Medical Sciences, Daejeon, Korea; 3 Department of Public Health, Graduate School, Chungnam National University, Daejeon, Korea

**Keywords:** Usual source of care, Continuity of care, Diabetes mellitus, Medication adherence, Health behavior

## Abstract

**OBJECTIVES:**

This study investigated the associations between usual source of care (USC) characteristics, which incorporate primary care functions, and medication adherence and healthy behaviors in Korean adults with diabetes.

**METHODS:**

We used data from 1,543 adults with diabetes in the 2020 Korea Health Panel Survey. USC was categorized into 5 types based on whether a regular doctor was identified and whether that provider fulfilled comprehensiveness and coordination functions. Multivariable logistic regression was used to assess associations with medication adherence and healthy behaviors.

**RESULTS:**

A significant difference in medication adherence was observed by USC type, although no significant associations emerged for healthy behaviors (smoking, drinking, exercise). Compared to the group without a USC, patients whose regular doctor fulfilled either function were 2.38 times more likely to adhere (odds ratio [OR], 2.38; 95% confidence interval [CI], 1.70 to 3.32), and those whose doctor fulfilled both functions were 1.84 times more likely to adhere (OR, 1.84; 95% CI, 1.31 to 2.59). This association was particularly strong for adherence to medication dosage, frequency, and timing.

**CONCLUSIONS:**

The findings underscore that the functional quality of the USC, particularly the fulfillment of comprehensiveness and coordination, is crucial in improving medication adherence. Simply having a USC is insufficient. The lack of association with healthy behaviors suggests that physicians may focus more on pharmacological control, highlighting the need for multifaceted interventions.

## GRAPHICAL ABSTRACT

[Fig f1-epih-47-e2025063]

## Key Message

This study shows that simply having a usual source of care (USC) is insufficient; only USCs that fully perform primary care functions are significantly associated with better medication adherence in diabetic patients. However, USC was not linked to improved health behaviors, suggesting providers fail to influence lifestyle changes. The findings provide policy evidence to reinforce the primary care functions of USCs to enhance chronic disease management.

## INTRODUCTION

Diabetes mellitus imposes a significant disease burden worldwide [1,2]. In Korea, approximately 5.57 million adults aged 19 years and older—about 12.5% of the population—are estimated to have diabetes. Among both men and women, diabetes accounts for the largest number of disability-adjusted life years lost, making it the most burdensome chronic disease [3]. Maintaining good glycemic control is essential for reducing this burden [4]. Nevertheless, previous studies have shown that more than half of individuals with diabetes exhibit poor glycemic control [2,4,5]. Glycosylated hemoglobin (HbA1c) is a key indicator for evaluating glycemic control, yet in Korea, only 32.4% of patients achieve HbA1c levels below 6.5% [6]. Poor glycemic control is reportedly influenced by low medication adherence [2,7] and unhealthy behaviors, including insufficient physical activity or smoking [2,8,9].

To improve poor glycemic control in patients with diabetes, both inadequate medication adherence and unhealthy lifestyle behaviors must be addressed; therefore, the usual source of care (USC) is expected to play a central role. The USC refers to a healthcare provider or institution that patients primarily visit when they are ill [10]. In chronic diseases requiring continuous management and treatment, the role of healthcare providers is especially important in encouraging patients to adopt behaviors beneficial for disease control in their daily lives. When the USC operates effectively, it is expected to generate positive outcomes in diabetes management [11,12]. In countries such as the United Kingdom, the USC contributes to the continuity and sustainability of care by promoting long-term patient-provider relationships. These providers serve as the first point of contact for new health concerns and play a central role in treatment, preventive services, and coordination of care, all of which are essential elements of primary care [10,13]. However, in Korea, the role of primary care is not firmly established within the healthcare delivery system. Patients can access private clinics as well as secondary and tertiary hospitals with minimal restrictions, which undermines the development of a consistent USC. In major developed countries such as the United States, Canada, Germany, the Netherlands, New Zealand, and the United Kingdom, more than 80-90% of the population reports having a USC [13,14]. In contrast, only 53.4% of Koreans reported having a USC in 2021, reflecting a comparatively low adoption rate [15]. As a result, critics argue that, unlike countries with a strong primary care orientation, Korea lacks consistent first-contact care and adequate guarantees of comprehensiveness or coordination due to the widespread establishment of specialty-based clinics [16].

Numerous studies have demonstrated that having a USC positively influences chronic disease management. Patients with a USC are more likely to receive preventive services, including routine checkups, than those without a regular source of care [10,17-19]. In the United States, previous research has shown that patients with a USC and high continuity of care actively engage in desirable self-care behaviors for diabetes management. These patients reportedly demonstrate higher treatment adherence, improved glycemic control, and reduced risks of complications [17,20,21]. In Korea, several recent studies have also reported higher medication adherence among diabetes patients with a USC [22,23]. These studies either defined USC broadly as having a regular doctor or place [22], or followed approaches from foreign literature by categorizing USC types into “regular doctor,” “place only,” and “no USC” [23]. However, considering that the USC in Korea does not fully perform the role of primary care [16], unlike in other countries, a more specific interpretation of the USC’s effects on diabetes management requires analyzing whether the regularly visited provider fulfills comprehensiveness and coordination functions. To date, no studies have incorporated these critical variables. Furthermore, although healthy behaviors are essential for glycemic control, to the best of our knowledge, very few studies have examined the association between the USC and healthy behaviors.

Accordingly, this study aims to investigate the associations between USC characteristics and both medication adherence and engagement in healthy behaviors (e.g., smoking cessation, alcohol abstinence, and physical activity) among adults with diabetes in Korea. The hypothesis of this study is that patients with diabetes whose USC fulfills both comprehensive and coordinating functions will demonstrate higher levels of medication adherence and a greater likelihood of participating in healthy behaviors.

## MATERIALS AND METHODS

### Data source

The data used in this study were obtained from the Korea Health and Social Research Institute and the National Health Insurance Corporation, which jointly produced the annual dataset (version 2.2) in Korea. The study sample included 1,543 adults aged 19 years or older who reported having diabetes in the 2020 Korea Health Panel Survey.

### Outcome definitions

The dependent variables in this study were medication adherence, current smoking, high-risk drinking, and physical activity. Medication adherence was assessed among patients prescribed diabetes medication using 3 questions: (1) “During the past year, did you take your prescribed medication as directed (number of tablets per dose)?” (2) “Did you follow the prescribed timing of medication intake (before meals, after meals, or at bedtime)?” and (3) “Did you take your medication as instructed in terms of method of administration?” In addition, participants were asked: “During the past year, have you ever discontinued your prescribed diabetes medication without consulting your physician?” Respondents who answered “No” to this final question were classified as demonstrating complete adherence [22]. Discontinuation of medication was analyzed separately.

Current smoking status was determined by the question: “Do you currently smoke cigarettes?” High-risk drinking was defined differently for men and women. Women were classified as high-risk drinkers if they reported consuming 5 or more drinks on a single occasion, and men if they reported 7 or more. Frequency was also considered; participants who indicated drinking “2-3 times/wk,” “≥4 times/wk,” or “almost daily” in the past year were categorized as high-risk drinkers. Additionally, the amount of alcohol consumed per occasion was assessed. Women who reported drinking “5-6 glasses,” “7-9 glasses,” or “10 or more glasses” per occasion were classified as high-risk drinkers. Physical activity was assessed based on whether respondents had engaged in exercise, including walking, during the past year. Participants were then categorized into 2 groups: the exercise group and the non-exercise group.

### Variables of interest

The USC was categorized into 5 types based on the presence of a regular doctor and the functional characteristics of that care, particularly comprehensiveness and coordination: (1) no USC, (2) place only (having a USC but no regular doctor), (3) regular doctor without comprehensiveness and coordination functions, (4) regular doctor with either comprehensiveness or coordination functions, and (5) regular doctor with both comprehensiveness and coordination functions. The questions asked were: “Do you have a medical institution (regular place) that you usually visit when you are sick or need a health checkup or treatment?” and “Do you have a regular doctor you usually visit when you are sick or need a checkup or treatment?” Responses for both were categorized as “yes” or “no.” To assess the comprehensiveness of the provider’s care, participants were asked: “Does that doctor address most of your common health problems?” Responses included: “almost always,” “generally,” “neutral,” “generally not,” and “not at all.” To evaluate the coordination function, respondents were asked: “Does that doctor appropriately refer you to other healthcare facilities or professionals when needed for your health care?” The same response scale was used: “almost always,” “generally,” “neutral,” “generally not,” and “not at all.”

### Covariates

The participants’ baseline socio-demographic factors included gender, age, education level, average monthly household income, marital status, health insurance, disability, subjective health status, and number of chronic diseases. Average monthly household income was calculated by dividing the total annual household income—comprising wages, business income, property income, and transfer income—by 12 months. The number of chronic conditions, excluding diabetes, was categorized into 4 groups: 0, 1, 2, and 3 or more.

### Statistical analysis

First, descriptive analyses were conducted to examine the distribution of private healthcare type and general characteristics, including gender, age, marital status, education level, type of health insurance, private medical insurance subscription, disability status, self-rated health, and the number of chronic diseases. Second, multivariable logistic regression analyses were performed to assess the associations of complete medication adherence, current smoking, high-risk drinking, and physical activity (dependent variables) with the independent variables. Third, additional multivariable logistic regression analyses were conducted to examine whether there were significant differences in medication adherence subcomponents (dosage, timing, and method of administration) among patients with diabetes, according to type of private healthcare source. A 2-sided significance level of p-value<0.05 was applied to all statistical analyses.

### Ethics statement

The Korea Health Panel Survey was approved by Statistics Korea pursuant to Article 18 of the Statistics Act (approval No. 920012). For this study, an exemption from review was granted by the institutional review board (IRB No. 202507-SB-130-01) of the university to which the researcher belongs.

## RESULTS

Regarding the general characteristics of the study participants, 76.4% of diabetic patients reported having a USC, with 23.7% belonging to the ‘place only’ group. Medication adherence was observed in 65.6% of the patients. For health behaviors, the current smoking rate was 15.1%, the high-risk drinking rate was 9.0%, and the physical activity participation rate was 50.5% (Table 1).

A statistically significant difference was observed in medication adherence according to USC type, whereas no significant differences were found in the practice rates of health behaviors. Patients whose USC provided both comprehensiveness and coordination functions showed a medication adherence rate of 72.0%, and those whose USC fulfilled either comprehensiveness or coordination demonstrated the highest adherence rate at 76.3%. In contrast, lower adherence rates were seen among patients without a regular medical institution (57.7%) and those without a regular physician (57.0%). No statistically significant differences were identified in the prevalence of smoking, high-risk alcohol use, or regular exercise across USC types (Table 2).

After adjustment for covariates, compared to the no USC group (the reference group), the regular doctor group with either comprehensiveness or coordination functions was 2.38 times more likely to report medication adherence (p<0.001), while the regular doctor group with both functions was 1.84 times more likely to adhere to medications (p<0.001). However, no statistically significant associations were observed between USC type and health behaviors such as smoking, high-risk alcohol use, or regular exercise (Table 3).

Additionally, multivariate logistic regression analysis was conducted to examine whether different types of USC among patients with diabetes were associated with various aspects of medication adherence, including correct dosage, dosage frequency, timing, and continuation of medication without interruption. The analysis compared all 5 USC types using the no USC group as the reference. For adherence to correct dosage, patients in the regular doctor group without comprehensiveness and coordination functions were 2.50 times more likely to adhere to the correct dose (p=0.001). Those in the regular doctor group with either comprehensiveness or coordination functions showed a 2.84 times higher likelihood of correct dosage adherence (p<0.001). For adherence to medication frequency, the regular doctor group without comprehensiveness and coordination functions was 1.85 times more likely to be adherent (p=0.017), while the regular doctor group with either comprehensiveness or coordination functions and the regular doctor group with both functions showed 2.47 times and 1.82 times higher odds, respectively. Regarding medication timing, patients in the regular doctor group with either comprehensiveness or coordination functions and the regular doctor group with both functions were significantly more likely to follow the correct schedule, with odds ratios of 2.60 and 1.82, respectively (both p<0.001). As for continuation of medication without interruption, although all USC types tended to show higher odds of adherence than the reference group, none of these associations reached statistical significance (Table 4).

## DISCUSSION

According to the findings, as of 2021, 76.4% of Korean adults with diabetes reported having a USC. When excluding those who relied solely on medical institutions without a regular physician, the proportion with a physician-based USC was 52.7%. Compared to the general Korean adult population, in which the USC rate was 53.4% [15], adults with diabetes were more likely to have a USC. However, when compared with the United States—where 94.8% of patients with diabetes have a USC and 85.5% have a regular physician [20]—the rate among Korean patients with diabetes remains relatively low. Furthermore, in this study, only 43.6% of patients with diabetes who had a USC reported that their provider fulfilled at least 1 of the 2 core primary care functions, comprehensiveness or coordination. This is substantially lower than in the United States, where approximately 95% of patients report receiving all 4 key primary care attributes—first-contact access, comprehensiveness, coordination, and continuity [12]. These findings suggest that although many patients with diabetes in Korea have a regular source of care, that source often fails to provide the core functions expected of primary care.

The USC in Korea frequently represents only a commonly visited healthcare facility, rather than reflecting the core attributes of primary care, as is the case in the United States and the United Kingdom. Consequently, there has been a sustained need for studies on USC that better capture the primary care concept [11,16,22]. Therefore, unlike previous studies that defined USC solely as having a commonly visited institution or physician [11,22,23], this study used an expanded definition that incorporated whether the regular physician performed comprehensiveness and coordination functions. This allowed us to identify meaningful variations in the association between USC type and medication adherence. Our findings showed that patients with a regular physician whose care included either comprehensiveness or coordination had approximately twice the medication adherence of those without a USC. However, no significant association was found when patients with diabetes had only a USC institution without a regular physician, or when they had a regular physician who did not perform these primary care functions. This pattern was further demonstrated in the sub-analysis of adherence components. Patients with a regular physician who fulfilled either comprehensiveness or coordination functions had higher odds of adherence not only to dosage and frequency but also to medication timing. These results indicate that a high-quality USC relationship is essential for patients to understand and practice the importance of medication adherence. In this regard, our findings address limitations in previous studies that did not consider the functional dimension of the USC. While earlier research reported that patients with diabetes who have a USC exhibit higher medication adherence than those without [17,20-23], our results indicate that a meaningful effect appears only when the USC delivers the core primary care attributes of comprehensiveness and coordination.

The positive effect of USC on medication adherence has generally been explained through the continuity of the patient–physician relationship. According to a systematic review, communication between patient and physician plays a central role in the quality of the USC relationship [24], and patient-centered communication increases patient trust and allows providers to incorporate patients’ preferences and values into treatment decisions [24,25]. This patient trust and shared decision-making can subsequently enhance adherence to treatment, ultimately leading to better health outcomes [25-27]. Prior studies have found that patients with type 2 diabetes who report poor communication with their providers show lower adherence to treatment recommendations and medication regimens than those who report better communication [25-29]. Providers who know their patients well due to continuity are also more confident in adjusting medication regimens, which can contribute to improved glycemic control. Continuity may further enhance adherence by increasing patient trust in their healthcare provider [21].

In this study, the USC among patients with diabetes was significantly associated with medication adherence, but no significant association was found with healthy behaviors. An additional analysis that classified USC medical institutions into primary care-level clinics and hospital-level facilities produced results consistent with the main findings, which did not distinguish between institution types. Specifically, the association between USC type and medication adherence remained significant in both clinic-level and hospital-level settings, while no significant association appeared for healthy behaviors. Although studies focusing specifically on patients with diabetes are limited, prior research examining the general population has shown mixed results regarding the effect of a USC on healthy behaviors. Findings consistently indicate no significant association between USC and smoking cessation [30-32], whereas other studies have reported positive associations between USC and physical activity [30] or alcohol consumption [31].

Several explanations have been proposed in the literature for these findings. First, even when continuity of care is present, primary care physicians may not place strong emphasis on preventive counseling for health behavior change. In clinical practice, particularly when consultation time is limited, behavioral counseling may be deprioritized in favor of immediate clinical concerns such as glycemic control [33]. Our findings suggest that while having a USC, especially one with comprehensive or coordinating functions, may improve medication adherence, it does not appear to significantly influence health behavior. This may indicate that physicians focus their efforts more on managing blood glucose levels through pharmacological treatment than on encouraging lifestyle modifications. Another possible explanation for the lack of association between having a USC and healthy behaviors is that, even when physicians provide counseling or information, patients may fail to retain the advice or follow through with the recommended actions [32]. Levine et al. [12] found that patients with diabetes who received primary care through a USC were more likely to receive counseling on smoking cessation, exercise, and weight management compared to those without a USC. However, this suggests that although USC providers may offer more frequent or comprehensive counseling, additional factors are likely required to translate advice into sustained behavioral change. Behaviors such as smoking and alcohol use—which involve personal preferences and elements of addiction—may be particularly resistant to change through counseling alone [30]. Moreover, individuals with diabetes are often less inclined to engage in moderate or vigorous physical activity than those without chronic conditions and tend to lead more sedentary lifestyles, spending a greater proportion of the day sitting [34]. These findings indicate that merely having a USC is insufficient to produce substantial improvements in health behaviors. Instead, they highlight the complexity of behavior change in chronic disease management and underscore the need for multifaceted, sustained interventions beyond routine clinical care.

This study has several limitations. First, because the data rely on self-reported responses, there may be discrepancies between reported medication adherence and actual adherence behaviors. Various methods for assessing medication adherence exist, including biochemical measures such as drug levels in blood or urine and objective methods such as calculating medication possession ratios based on prescription refill data [23]. However, this study relied on an indirect method—self-reported questionnaire data—which is subject to recall bias and social desirability bias, potentially affecting the accuracy of the findings. Second, this was a cross-sectional study based on data collected in 2020, making it difficult to establish clear causal relationships. Although the presence of a USC may influence medication adherence, it is also possible that patients with higher adherence are more likely to seek out or maintain a USC. Future prospective cohort studies are needed to better assess the causal impact of USC on both medication adherence and health behaviors.

Despite these limitations, this study differs from previous research by emphasizing not only the presence of a USC but also the quality of care provided, particularly regarding fulfillment of the core attributes of primary care—comprehensiveness and coordination. The findings suggest that USC can have a meaningful impact on diabetes management when it functions according to primary care principles, supporting both patients’ understanding of treatment and their appropriate medication-taking behavior.

## Figures and Tables

**Figure f1-epih-47-e2025063:**
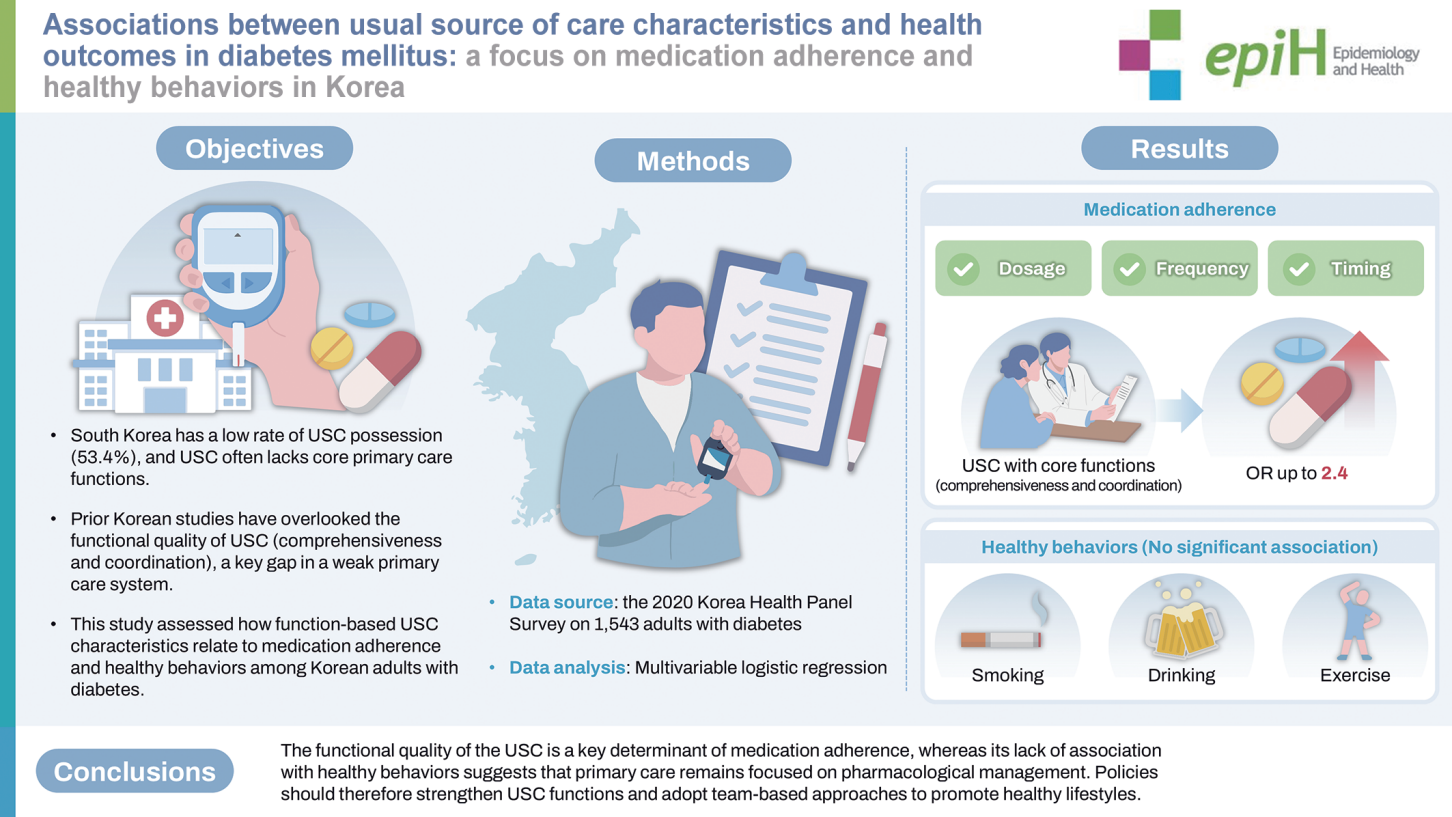


**Table 1.** Diabetes patients’ general characteristics in the second Korea Health Panel (2020) data (n=1,543)

Characteristics	n (%) or mean±SD	Characteristics	n (%) or mean±SD
Gender		1	512 (33.2)
Man	756 (49.0)	2	409 (26.5)
Woman	787 (51.0)	≥3	364 (23.6)
Age (yr)	69.06±10.84	OPD visits per year	27.25±27.20
Education level		USC type^3^	
Elementary school or less	590 (38.2)	No USC	358 (23.6)
Middle school	297 (19.2)	Place only	360 (23.7)
High school	413 (26.8)	Regular doctor, non-functional	139 (9.1)
University or higher	243 (15.7)	Regular doctor, partially functional	357 (23.5)
Marital status		Regular doctor, comprehensiveness only	14 (0.9)
Married	1,071 (69.4)	Regular doctor, coordination only	343 (22.6)
Others^1^	472 (30.6)	Regular doctor, fully functional	306 (20.1)
Average monthly household income (10^4^ KRW)	291.80±268.66	USC medical institutions^4^	
Health coverage		Clinic	714 (61.8)
NHI	1,420 (92.0)	Hospital	442 (38.2)
Others^2^	123 (8.0)	Medication adherence	
Private health insurance		Yes	977 (65.6)
Yes	903 (59.8)	No	512 (34.4)
No	606 (40.2)	Smoking status	
Disability		Yes	229 (15.1)
Yes	221 (14.3)	No	1,291 (84.9)
No	1,322 (85.7)	High-risk drinking	
Self-rated health status		Yes	132 (9.0)
Poor	288 (18.9)	No	1,340 (91.0)
Moderate	680 (44.7)	Physical activity	
Good	552 (36.3)	Yes	767 (50.5)
No. of other chronic diseases		No	753 (49.5)
0	258 (16.7)	Total	1,543 (100)

Data were missing for some respondents in the following characteristics: private health insurance, 34; self-rated health status, 23; USC type, 23; USC medical institutions, 387: OPD visits per year, 12; medication adherence, 54; smoking status, 23; high-risk drinking, 71; physical activity, 23. SD, standard deviation; KRW, Korean won; NHI, National Health Insurance; USC, usual source of care; OPD, outpatient department.

1 Other marital status groups: divorced, separated, widowed, and never married.

2 Other health coverage groups: all health insurance (medical care for low-income people, special care for national merit, care for foreigners, and stopped care [non-payment]) other than NHI.

3 No USC group; Place only group: having a USC institution but no regular doctor; Regular doctor (non-functional) group: regular doctor group without comprehensiveness and coordination functions; Regular doctor (partially functional) group: regular doctor group with either comprehensiveness or coordination functions; This group includes regular doctor, comprehensiveness only group and regular doctor, coordination only group; Regular doctor (fully functional) group: regular doctor group with both comprehensiveness and coordination functions.

4 Clinic: clinic, public health center, public health subcenter, primary health care post; Hospital: hospital, general hospital. traditional Korean medicine hospitals and clinics were missing.

**Table 2.** Differences in medication adherence and health behaviors by general characteristics

Variables	Medication adherence (n=1,489)			Smoking status (n=1,520)			High-risk drinking (n=1,472)			Physical activity (n=1,520)		
	No	Yes	p-value^1^	No	Yes	p-value^1^	No	Yes	p-value^1^	No	Yes	p-value^1^
Gender			0.258			<0.001			<0.001			<0.001
Man	241 (33.0)	490 (67.0)		535 (71.9)	209 (28.1)		605 (82.8)	126 (17.2)		335 (45.0)	409 (55.0)	
Woman	271 (35.8)	487 (64.2)		756 (97.4)	20 (2.6)		735 (99.2)	6 (0.8)		418 (53.9)	358 (46.1)	
Age (yr)	68.81±11.85	69.16±10.22	0.573	69.93±10.63	63.55±10.40	<0.001	69.56±10.72	62.61±9.68	<0.001	68.78±11.20	69.14±10.46	0.516
Education level			0.890			<0.001			<0.001			<0.001
Elementary school or less	200 (35.4)	365 (64.6)		527 (91.7)	48 (8.3)		529 (95.1)	27 (4.9)		326 (56.7)	249 (43.3)	
Middle school	96 (33.0)	195 (67.0)		253 (86.1)	41 (13.9)		261 (93.5)	18 (6.5)		146 (49.7)	148 (50.3)	
High school	138 (34.6)	261 (65.4)		320 (77.9)	91 (22.1)		355 (87.2)	52 (12.8)		194 (47.2)	217 (52.8)	
University or higher	78 (33.3)	156 (66.7)		191 (79.6)	49 (20.4)		195 (84.8)	35 (15.2)		87 (36.3)	153 (63.7)	
Marital status			0.253			0.846			0.014			<0.001
Married	348 (33.5)	692 (66.5)		899 (85.1)	158 (14.9)		917 (89.8)	104 (10.2)		492 (46.5)	565 (53.5)	
Others^2^	164 (36.5)	285 (63.5)		392 (84.7)	71 (15.3)		423 (93.8)	28 (6.2)		261 (56.4)	202 (43.6)	
Average monthly household income (10^4^ KRW)	269.00±214.46	304.86±295.42	0.007	285.66±270.71	331.79±262.83	0.017	280.38±258.24	429.42±356.94	<0.001	288.43±250.58	296.71±287.81	0.550
Health coverage			0.667			0.105			0.071			0.035
NHI	470 (34.2)	903 (65.8)		1,196(85.4)	205 (14.6)		1,230 (90.6)	127 (9.4)		683 (48.8)	718 (51.2)	
Others^3^	42 (36.2)	74 (63.8)		95 (79.8)	24 (20.2)		110 (95.7)	5 (4.3)		70 (58.8)	49 (41.2)	
Private health insurance			0.704			<0.001			0.017			0.231
Yes	300 (34.1)	581 (65.9)		745(82.5)	158 (17.5)		781 (89.6)	91 (10.4)		435 (48.2)	468 (51.8)	
No	209 (35.0)	388 (65.0)		538 (88.8)	68 (11.2)		549 (93.2)	40 (6.8)		31 1(51.3)	295 (48.7)	
Disability			0.772			0.037			0.031			0.010
Yes	69 (33.5)	137 (66.5)		191 (89.7)	22 (10.3)		192 (95.0)	10 (5.0)		123 (57.7)	90 (42.3)	
No	443 (34.5)	840 (65.5)		1,100 (84.2)	207 (15.8)		1,148 (90.4)	122 (9.6)		630 (48.2)	677(51.8)	
Self-rated health sta	tus		<0.001			0.012			<0.001			<0.001
Poor	85 (30.5)	194 (69.5)		256 (88.9)	32 (11.1)		239 (85.4)	41 (14.6)		99 (34.4)	189 (65.6)	
Moderate	206 (31.0)	458 (69.0)		558 (82.1)	122 (17.9)		600 (90.4)	64 (9.6)		314 (46.2)	366 (53.8)	
Good	221 (40.5)	325 (59.5)		477 (86.4)	75 (13.6)		501 (94.9)	27 (5.1)		340 (61.6)	212 (38.4)	
No. of other chronic diseases			0.019			<0.001			<0.001			
0	90 (36.6)	156 (63.4)		202 (78.9)	54 (21.1)		214 (87.3)	31 (12.7)		101 (39.5)	155 (60.5)	
1	145 (29.6)	345 (70.4)		407 (80.8)	97 (19.2)		418 (85.5)	71 (14.5)		228 (45.2)	276 (54.8)	
2	136 (34.2)	262 (65.8)		352 (87.8)	49 (12.2)		365 (93.6)	25 (6.4)		217 (54.1)	184 (45.9)	
≥3	141 (39.7)	214 (60.3)		330 (91.9)	29 (8.1)		343 (98.6)	5 (1.4)		207 (57.7)	152 (42.3)	
OPD visits per year	27.40±27.45	27.25±26.78	0.920	28.07±27.64	22.62±23.62	0.002	28.22±28.11	17.83±15.32	<0.001	28.54±29.85	25.99±24.14	0.069
USC type^4^			<0.001			0.304			0.757			0.984
No USC	146 (42.3)	199 (57.7)		306 (85.5)	52 (14.5)		318 (91.9)	28 (8.1)		177 (49.4)	181 (50.6)	
Place only	153 (43.0)	203 (57.0)		297 (82.5)	63 (17.5)		313 (89.7)	36 (10.3)		183 (50.8)	177 (49.2)	
Regular doctor, non-functional	46 (33.3)	92 (66.7)		115 (82.7)	24 (17.3)		123 (91.1)	12 (8.9)		67 (48.2)	72 (51.8)	
Regular doctor, partially functional	83 (23.7)	267 (76.3)		314 (88.0)	43 (12.0)		318 (92.2)	27 (7.8)		176 (49.3)	181 (50.7)	
Regular doctor, fully functional	84 (28.0)	216 (72.0)		259 (84.6)	47 (15.4)		268 (90.2)	29 (9.8)		150 (49.0)	156 (51.0)	

Values are presented as number (%). KRW, Korean won; NHI, National Health Insurance; USC, usual source of care; OPD, outpatient department.

1 Using chi-square test and t-test.

2 Other marital status groups: divorced, separated, widowed, and never married.

3 Other health coverage groups: all health insurance (medical care for low-income people, special care for national merit, care for foreigners, and stopped care [non-payment]) other than NHI.

4 USC type = No USC group; place only group: having a USC institution but no regular doctor; regular doctor (non-functional) group: regular doctor group without comprehensiveness and coordination functions; regular doctor (partially functional) group: regular doctor group with either comprehensiveness or coordination functions; This group includes regular doctor, comprehensiveness only group and regular doctor, coordination only group. regular doctor (fully functional) group: regular doctor group with both comprehensiveness and coordination functions.

**Table 3.** Factors associated with medication adherence and health behaviors

Variables	Medication adherence	Smoking status	High-risk drinking	Physical activity
Gender				
Man	1.00 (reference)	1.00 (reference)	1.00 (reference)	1.00 (reference)
Woman	1.04 (0.81, 1.34)	22.10 (13.09, 37.32)	24.09 (10.21, 56.81)	0.98 (0.77, 1.24)
Age (yr)	1.01 (1.00, 1.02)	1.05 (1.03, 1.07)	1.06 (1.03, 1.08)	1.02 (1.01, 1.04)
Education level				
Elementary school or less	1.00 (reference)	1.00 (reference)	1.00 (reference)	1.00 (reference)
Middle school	1.09 (0.79, 1.51)	1.07 (0.64, 1.79)	1.57 (0.79, 3.09)	1.22 (0.90, 1.65)
High school	0.94 (0.69, 1.29)	0.98 (0.61, 1.57)	1.41 (0.79, 2.53)	1.40 (1.04, 1.90)
University or higher	0.93 (0.62, 1.39)	1.50 (0.84, 2.69)	2.48 (1.21, 5.05)	2.29 (1.55, 3.38)
Marital status				
Married	1.00 (reference)	1.00 (reference)	1.00 (reference)	1.00 (reference)
Others^1^	1.00 (0.77, 1.31)	0.47 (0.31, 0.71)	0.79 (0.46, 1.34)	0.80 (0.62, 1.03)
Average monthly household income (10^4^ KRW)	1.00 (1.00, 1.00)	1.00 (1.00, 1.00)	1.00 (1.00, 1.00)	1.00 (1.00, 1.00)
Health coverage				
NHI	1.00 (reference)	1.00 (reference)	1.00 (reference)	1.00 (reference)
Others^2^	1.12 (0.71, 1.74)	0.61 (0.31, 1.17)	1.57 (0.52, 4.69)	0.91 (0.60, 1.40)
Private health insurance				
Yes	1.00 (reference)	1.00 (reference)	1.00 (reference)	1.00 (reference)
No	1.08 (0.83, 1.40)	1.46 (0.99, 2.15)	0.85 (0.53, 1.37)	0.90 (0.70, 1.15)
Disability				
Yes	1.00 (reference)	1.00 (reference)	1.00 (reference)	1.00 (reference)
No	0.82 (0.59, 1.16)	0.47 (0.27, 0.84)	0.70 (0.34, 1.46)	1.25 (0.91, 1.72)
Self-rated health status				
Poor	1.00 (reference)	1.00 (reference)	1.00 (reference)	1.00 (reference)
Moderate	1.04 (0.76, 1.42)	0.40 (0.25, 0.65)	1.55 (0.96, 2.51)	0.66 (0.49, 0.89)
Good	0.67 (0.48, 0.94)	0.34 (0.20, 0.59)	1.60 (0.87, 2.91)	0.39 (0.28, 0.54)
No. of other chronic diseases				
0	1.00 (reference)	1.00 (reference)	1.00 (reference)	1.00 (reference)
1	1.38 (0.98, 1.95)	0.97 (0.62, 1.51)	0.54 (0.32, 0.91)	0.75 (0.54, 1.04)
2	1.06 (0.73, 1.55)	0.99 (0.58, 1.69)	0.69 (0.35, 1.35)	0.62 (0.43, 0.89)
≥3	0.94 (0.62, 1.41)	1.19 (0.63, 2.27)	1.92 (0.65, 5.66)	0.61 (0.41, 0.90)
OPD visits per year	1.00 (1.00, 1.01)	1.01 (1.00, 1.01)	1.02 (1.00, 1.03)	1.00 (1.00, 1.01)
USC type^3^				
No USC	1.00 (reference)	1.00 (reference)	1.00 (reference)	1.00 (reference)
Place only	0.95 (0.70, 1.29)	0.74 (0.46, 1.19)	0.66 (0.37, 1.19)	1.02 (0.75, 1.39)
Regular doctor, non-functional	1.42 (0.93, 2.17)	0.90 (0.49, 1.69)	0.86 (0.39, 1.86)	1.04 (0.69, 1.57)
Regular doctor, partially functional	2.38 (1.70, 3.32)	1.05 (0.63, 1.74)	0.77 (0.42, 1.43)	1.05 (0.77, 1.44)
Regular doctor, fully functional	1.84 (1.31, 2.59)	1.10 (0.66, 1.82)	0.85 (0.46, 1.57)	1.03 (0.74, 1.42)

Values are presented as adjusted odds ratio (95% confidence interval). KRW, Korean won; NHI, National Health Insurance; USC, usual source of care; OPD, outpatient department.

1 Other marital status groups: divorced, separated, widowed, and never married.

2 Other health coverage groups: all health insurance (medical care for low-income people, special care for national merit, care for foreigners, and stopped care [non-payment]) other than NHI.

3 No USC group; Place only group: having a USC institution but no regular doctor; Regular doctor (non-functional) group: regular doctor group without comprehensiveness and coordination functions; Regular doctor (partially functional) group: regular doctor group with either comprehensiveness or coordination functions; This group includes regular doctor, comprehensiveness only group and regular doctor, coordination only group; Regular doctor (fully functional) group: regular doctor group with both comprehensiveness and coordination functions.

**Table 4.** Association between usual source of care (USC) type and subcomponents of medication adherence (dosage, frequency, timing, and continuation)^1^

USC type^2^	Dosage	Frequency	Timing	Continuation
No USC	1.00 (reference)	1.00 (reference)	1.00 (reference)	1.00 (reference)
Place only	1.28 (0.91, 1.82)	1.16 (0.83, 1.63)	0.97 (0.71, 1.32)	1.98 (0.95, 4.14)
Regular doctor, non-functional	2.50 (1.43, 4.35)	1.85 (1.12, 3.07)	1.31 (0.85, 2.00)	2.19 (0.73, 6.57)
Regular doctor, partially functional	2.84 (1.89, 4.27)	2.47 (1.67, 3.63)	2.60 (1.84, 3.68)	1.15 (0.60, 2.21)
Regular doctor, fully functional	1.99 (1.33, 2.97)	1.82 (1.24, 2.68)	1.82 (1.29, 2.58)	1.51 (0.72, 3.17)

Value are presented as adjusted odds ratio (95% confidence interval).

1 Adjusted for gender, age, education level, marital status, average monthly household income, health coverage, private health insurance, disability, self-rated health status, the number of other chronic diseases, and outpatients visits per year; Hosmer–Lemeshow goodness-of-fit: all models fit well.

2 No USC group; Place only group: having a USC institution but no regular doctor; Regular doctor (non-functional) group: regular doctor group without comprehensiveness and coordination functions; Regular doctor (partially functional) group: regular doctor group with either comprehensiveness or coordination functions; This group includes regular doctor, comprehensiveness only group and regular doctor, coordination only group; Regular doctor (fully functional) group: regular doctor group with both comprehensiveness and coordination functions.
